# Red Pepper (*Capsicum annuum* L.) Seed Extract Improves Glycemic Control by Inhibiting Hepatic Gluconeogenesis via Phosphorylation of FOXO1 and AMPK in Obese Diabetic *db*/*db* Mice

**DOI:** 10.3390/nu12092546

**Published:** 2020-08-23

**Authors:** Hyun Kyung Kim, Jeongho Jeong, Eun Young Kang, Gwang-woong Go

**Affiliations:** Department of Food and Nutrition, Hanyang University, Seoul 04763, Korea; hyunkyung@hanyang.ac.kr (H.K.K.); wjdgh7913@hanyang.ac.kr (J.J.); eunyoung94@hanyang.ac.kr (E.Y.K.)

**Keywords:** AMPK, FOXO1, glucose 6 phosphatase, gluconeogenesis, red pepper seed extract

## Abstract

Obesity is a notable risk factor for developing type 2 diabetes, augmenting the concern of obese diabetes (ObD). Anti-obesity and antioxidant effects of red pepper seeds extract (RPSE) have increased our expectations that RPSE would also improve the pathological phenotypes of obese diabetes. Therefore, we hypothesized that RPSE would have an anti-diabetic effect in ObD mice. Animals were assigned either as follows: (1) *db*/+, (2) *db/db* control, (3) RPSE (200 mg/kg bw), or (4) a comparative control (metformin 150 mg/kg bw). RPSE was orally administered daily for 8 weeks. As a result, RPSE supplementation improved diabetic phenotypes, including fasting glucose, hemoglobin (HbA1c), and insulin levels. Pro-inflammatory cytokines, tumor necrosis factor-alpha (TNF-α) and interleukin 6 (IL-6), and triglycerides were reduced in RPSE-treated mice. RPSE supplementation also diminished the rate-limiting enzymes of gluconeogenesis, including glucose 6-phosphatas (G6Pase) and phosphoenolpyruvate carboxykinase (PEPCK), in the liver. RPSE supplementation increased the phosphorylation of forkhead box protein O1 (FOXO1) and AMP-activated protein kinase (AMPK), which underlined the mechanism of the anti-diabetic effects of RPSE. Taken together, RPSE has the potential to improve glycemic control by repressing hepatic gluconeogenesis via the phosphorylation of FOXO1 and AMPK in ObD mice.

## 1. Introduction

Type 2 diabetes mellitus (T2DM) is a metabolic disease that is principally caused by impaired insulin signaling and the discontinuation of insulin production in severe cases [1,2]. T2DM is associated with serious complications, including dyslipidemia, retinopathy, neuropathy, kidney failure, and stroke [3]. The International Diabetes Federation estimated that approximately 415 million adults (age, 20–79 years) to have diabetes in 2015, and the number will rise up to 642 million by 2040 [4]. We need to focus on the concomitant rise of obesity and type 2 diabetes—obese diabetes (ObD). Obesity is an independent risk factor for developing T2DM; in fact, >90% of T2DM are overweight or obese [5]. It is profoundly established that the pathological mechanism of obesity, in particular insulin resistance, expedites the development of T2DM.

Red pepper (*Capsicum annuum* L.) has been adopted in several cuisines as a food additive worldwide [6]. Red pepper is not only a rich source of vitamin C, E, and carotenoids [7] but it also contains bioactive compounds including carotenoids (capsanthin, capsorubin, and carotenes), tocopherols, luteolin, quercetin, and capsaicin [8,9,10]. Previous studies on red pepper identified various beneficial physiological activities including anti-obesity, antioxidant, and anti-tumorigenesis [11,12,13]. Although studies on red pepper pulp have been actively undertaken, its seeds have been merely considered a by-product with little efficacy and value. In a recent study, red pepper seeds reduced lipid accumulation in vitro by inhibiting the expression of important adipogenic transcription factors including CCAAT/enhancer-binding protein α (C/EBPα) and peroxisome proliferator-activated receptor γ (PPARγ) in 3T3-L1 adipocytes [14]. Red pepper seeds also have an anti-mutagenic effect by increasing radical scavenging as an antioxidant agent [15]. Red pepper seeds reduced oxidative damage by the activation of anti-oxidative defense systems, including superoxide dismutase and catalase in rats fed with high-fat diets (HFDs) [16]. The physiological benefits of red pepper seeds mentioned above are not associated with capsaicin, which is seldom detected in red pepper seeds [17]. Instead, it has been recently identified that icariside E_5_ in red pepper seeds provided its physiological benefits [18].

As red pepper seeds extract (RPSE) exerts a potent antioxidant and anti-obesity effect, it is assumed to have anti-diabetic potential in obese diabetes. Nevertheless, the anti-diabetic effect of RPSE has not been clearly examined. Therefore, as an expansion to previous investigations, we hypothesized that dietary supplementation with RPSE improved the glycemic control of diabetes in ObD mice. To this end, *db/db* male mice were orally administered RPSE. Following the assessment of ObD phenotypes rescue, blood biochemistry and molecular pathways were explored to investigate the operative mechanisms.

## 2. Materials and Methods

### 2.1. Animals and Diets

RPSE was provided by Novarex, Inc. (Seoul, Korea), and it was prepared as previously described [19]. In brief, the company purchased the red pepper seed cultivated in Yeong-yang from Yeong Yang Red Pepper Trade Corp (Korea). The red pepper seed was blended with water (solid/water, 1:9) at 50 °C for 18–24 h followed by filtration (90 mm diameter, 5 μm pore size) (Hyundai Micro Co., Ltd., Seoul, Korea). The filtered extracts were concentrated using a vacuum evaporator at 40–50 °C and then spray-dried at 180–210 °C using a spray dryer (MSD-60-N; Niro Korea, Inc., Cheonan, Korea). In a previous study, icariside E_5_ (0.28%) and vanilloyl icariside E_5_ (0.085%) in RPSE, which were used in the current study, were quantitatively analyzed as standard substances [18].

All the procedures of the animal experiment were approved by Kookmin University IACUC (KMU-2016-7). Five-week-old male C57BL/KsJ *db/db* mice and their lean heterozygote littermates (*db*/+) were purchased from Daehan BioLink (Eumseong, Korea). The animal facility was maintained at 22 ± 1 °C and 50 ± 10% relative humidity, with a 12 h light/dark cycle. Four mice per cage housed in a ventilated cage were acclimatized for 1 week prior to experimentation. Then, mice were randomly divided into four groups: (1) heterozygote *db*/+, (2) *db/db* negative control (CTL), (3) RPSE (200 mg/kg bw), or (4) metformin (150 mg/kg bw). The experimental dose was decided based on a previous study, in which 200 mg/kg bw of RPSE suppressed HFD-induced obesity in mice [20]. Metformin was used as a comparative control (provided by Withus pharmaceutical, Anseong, Korea). The negative control was gavaged-only vehicle (distilled water). The materials were orally administered daily for 8 weeks. All mice were fed a normal chow diet (Harlan Laboratories Inc., Indianapolis, IN, USA), and food and water were provided *ad libitum*. The normal chow diet comprised 3.1 kcal/g (58% kcal from carbohydrate, 24% kcal from protein, and 18% kcal from fat). Body weight and food intake were measured weekly.

### 2.2. Fasting Blood Glucose and Oral Glucose Tolerance Test (OGTT)

Fasting blood glucose levels were measured every 2 weeks from the tail vein using a blood glucose test meter (Accuchek, Roche Diagnostics, Basel, Switzerland). The OGTT was performed at 6 weeks, and blood glucose levels were repeatedly measured at 0 (prior to glucose oral administration), 30, 60, and 120 min after glucose oral administration (2 g/kg bw). The areas under the curve (AUCs) of OGTT were calculated using Prism 6 (GraphPad Software, La Jolla, CA, USA).

### 2.3. Body Composition

Mice fasted for 4 h were anesthetized using ketamine (100 mg/kg bw) and xylazine (10 mg/kg bw) via intraperitoneal injection. Body composition, including fat in tissue percentage, lean body mass, and bone mineral density, was assessed using dual-energy X-ray absorptiometry (DEXA; Inalyzer, Medikors, Sungnam, Korea).

### 2.4. Blood and Tissue Sampling

The whole blood of 4-h fasted mice was obtained from the retro-orbital cavity using heparin-treated tubes. Plasma was collected by centrifugation (2000× *g*, 15 min, 4 °C). After sacrifice, liver tissue was snap-frozen and stored at −80 °C until the further determination of mRNA and protein expression.

### 2.5. Blood Biomarkers Assay

Plasma levels of alanine aminotransferase (ALT) and aspatate aminotransferase (AST) were measured spectrophotometrically using a commercial kit (Asan Co., Seoul, Korea) to determine hepatotoxicity. Concentrations of triglyceride (TG) and non-esterified fatty acid (NEFA) were measured using a commercial kit (Wako Ltd., Osaka, Japan). The plasma insulin was assayed using an ELISA kit (Morinaga Institute of Biological Science Inc., Yokohama, Japan). Glycated hemoglobin (HbA1c) was determined using a mouse hemoglobin A1c ELISA kit (Bioassay Technology Laboratory, Shanghai, China). Tumor necrosis factor-alpha (TNF-α) and interleukin 6 (IL-6) were estimated using ELISA kits (eBioscience, San Diego, CA, USA).

### 2.6. Quantitative RT-PCR

Total mRNA was isolated from liver tissue using Isol-RNA Lysis Reagent (5 PRIME Inc., Gaithersburg, MD, USA). The concentrations of total mRNA extracts were measured using NanoDrop One (Thermo Fisher Scientific, Waltham, MA, USA), and an iso-concentration was adapted. cDNA was prepared by the reverse-transcription of total mRNA using High Capacity RNA-to-cDNA Kit (Applied Biosystems, Foster City, CA, USA). qRT-PCR was performed in duplicates using the Taqman gene expression assay (Applied Biosystems). G6Pase (Mm00839363_m1) and PEPCK (Mm01247058_m1) were used, and 18 S (Mm04277571_m1) was used as a reference control. The quantification of mRNA levels was presented as cycle threshold values.

### 2.7. Immunoblotting

Tissue lysates were prepared using radioimmunoprecipitation assay buffer (CellNest, Tokyo, Japan) containing phosphatase and protease inhibitor (Cell Signaling Technology, Danvers, MA, USA). Total protein was determined by the Bradford method with bovine serum albumin (BSA) as the standard. Extracted protein was separated on 10% SDS-PAGE and transferred onto a polyvinylidene fluoride (PVDF) membrane. The membrane was blocked in 5% bovine serum albumin (GenDEPOT Inc., Barker, TX, USA) and immunoblotted using target primary antibodies. The blots were incubated in appropriate horseradish peroxidase (HRP)-conjugated secondary antibodies and quantified using Image Lab (Bio-Rad Co., Hercules, CA, USA). Antibodies to AMP-activated protein kinase (AMPK), phospho-AMPK (Thr172), forkhead box protein O1 (FOXO1), phospho-FOXO1 (Ser256), and β-actin were purchased from Cell Signaling Technology. Antibodies to phosphoenolpyruvate carboxykinase (PEPCK) and glucose 6-phosphatase (G6Pase) were purchased from Santa Cruz Biotechnology (Dallas, TX, USA).

### 2.8. Statistical Analysis

All data are presented as the mean ± SEM. Data were analyzed using a one-way analysis of variance, followed by Tukey’s multiple comparison analysis using Prism 6. A probability value of *p* < 0.05 was considered significant.

## 3. Results

### 3.1. RPSE Did Not Alter Growth Performance or Body Composition in ObD Mice

In the current study, the rodent model of obese diabetes was established by using *db/db* strain mice. According to the literature [21,22], *db/db* mice are characterized by insufficient leptin function (leptin resistance), which leads to the increase of feed intake, body weight, and fat accumulation. Excessive ectopic fat accumulation in *db/db* mice worsens insulin resistance by interfering with protein kinase B (PKB/AKT) kinase and glucose transporter 4 (GLUT4) translocation, which consequently leading to T2DM [23].

Prior to verifying the functionality of RPSE, a successful ObD model was established, and its rescue was confirmed by examining the (–) control and comparative control, metformin (Table 1 and Table 2). Characteristics of the growth performance of experimental animals are presented in Table 1. The final body weight (165.1%), weight gain (229%), and total energy intake (96.5%) in *db/db* (–) control mice increased compared to those of the *db*/+ (*p* < 0.05). Besides, *db/db* mice showed higher fat in tissue (113.2%) and lower lean body mass (13.2%) than *db*/+ (*p* < 0.05). In addition to obese phenotypes, the induction of T2DM including fasting glucose, HbA1c, and insulin levels in *db/db* mice has been verified in Table 2. *db/db* mice showed higher fasting glucose (289.3%), HbA1c (122.1%), and insulin (92.8%) levels than *db*/+ (*p* < 0.001). These results indicate that we successfully induced ObD phenotypes in *db/db* mice. Furthermore, the comparative drug, metformin, rescued pathological phenotypes, including body weight, fat in tissues, and blood glucose levels, as expected. Metformin is a first-line anti-diabetic agent that not only inhibits gluconeogenesis in the peripheral tissues [24,25] but also reduces body weight by suppressing lipid accumulation and appetite [26,27].

Next, the effects of RPSE on growth performance and body composition in ObD mice were assessed (Table 1). RPSE supplementation did not significantly alter body weight or tissue fat levels in ObD mice. A previous study has revealed similar results in that dried red pepper powder did not modulate weight gain or feed intake in Western diet (high fat and high cholesterol)-fed Sprague–Dawley rats [28]. As the reference study applied dried red pepper powder with the Western diet model rather than the solvent extract, there is a limit to securing the treatment materials’ compatibility. Another study showed that the capsicoside G-rich fraction from red pepper seeds (10 mg/kg bw) had no effect on adiposity in HFD-induced obese mice [29].

Our findings are contrary to expectations, because a recent study reported that RPSE hindered the increase in body weight and adiposity in HFD-induced obese mice by the inhibition of adipogenesis (via C/EBPα and PPARγ) of exogenous fat into white adipose tissues (WAT) [20]. The differences in these results are attributed to the fact that pathologic mechanisms of ObD mice, which are accompanied by leptin and insulin resistance, are not solely identical to those of the high fat-induced obesity model. In brief, *de novo* lipogenesis in the liver accelerated in *db/db* mice compared to the high-fat diet mice [30]. Overall, RPSE supplementation suggests that some differences exist depending on the experimental model; however, in *db/db* mice, RPSE does not modify body weight or fat accumulation.

### 3.2. RPSE Improved Risk Factors for Type 2 Diabetes Mellitus and Lipid Profiles in ObD Mice

The outcomes of RPSE supplementation in relevant risk factors of T2DM are presented in Table 2. The increased fasting glucose, HbA1c, and insulin levels (all *p* < 0.001) in *db/db* mice indicate a successful induction of hyperglycemia and hyperinsulinemia in the ObD model. These pathophysiological phenotypes were rescued by metformin; TG and NEFA levels reduced by 64% and 54%, respectively (all *p* < 0.001). RPSE supplementation diminished fasting blood glucose levels by 21% compared to those in the *db/db* control (*p* < 0.001). RPSE also distinctly decreased the level of HbA1c (22%), which is a long-term indicator of glycemic control to reveal the aggregate glycemic histories of the past 2–3 months [31]. In addition, the plasma insulin level was 29% lower in the RPSE group than in the control (p < 0.001). Similar to these findings, the anti-diabetes effect of red peppers has been reported by several preclinical and clinical intervention studies, in which its potency was identified mostly by the efficacy of capsaicin [32,33]. For instance, capsaicin treatment notably decreased fasting glucose levels in pancreatectomized diabetic rats, which is a moderate diabetic animal model [34]. Fasting glucose levels were recovered by 0.015% capsaicin for 4 weeks in alloxan-induced diabetic rats fed with a HFD [33]. Treatment with 5 mg/dL capsaicin for 4 weeks improved postprandial hyperglycemia and hyperinsulinemia in a randomized double-blind trial on gestational diabetes mellitus subjects [35]. Studies on capsaicin-independent anti-diabetes effects have recently reported that icariside E_5_-rich RPSE (100 and 200 mg/kg bw) reduced fasting glucose levels, insulin, and homeostatic model assessment of insulin resistance (HOMA-IR) in high-fat-induced obesity mice [18,20]. Our findings additionally highlight the anti-diabetic potency of RPSE in the ObD model, which has yet to be reported [20].

RPSE successfully improved T2DM diagnosis indicators; therefore, we further assessed insulin-resistance-associated biomarkers, including TG and NEFA (Table 2). *db/db* mice showed the development of hypertriglyceridemia and increased levels of NEFA compared to the *db*/+ mice, and these disease phenotypes all recovered to normal levels in the metformin group (all *p* < 0.001). RPSE supplementation prominently diminished the level of TG by 23% compared to the *db/db* control (*p* < 0.001). Likewise, a previous study has suggested that RPSE is capable of improving blood lipid profiles; the supplementation of red pepper seed ethanol extract (1–5 g/kg diet for 3 weeks) dose-dependently decreased blood TG and glucose levels in Sprague–Dawley rats [36]. NEFA from uncontrolled lipolysis can interfere with several adverse metabolic effects, most distinctly insulin resistance [37]. In this study, NEFA levels were reduced by 21% in RPSE-treated mice compared to those in the *db/db* control; however, their statistical significance was not verified. Regarding the impact of RPSE on NEFA, limited evidence is obtained, and further research concerning lipolysis-associated insulin resistance is required. Collectively, RPSE was identified as a potent anti-diabetic agent that ameliorates the biomarkers of diabetes diagnosis in the ObD model. The detailed mechanism of action linked to the anti-diabetes effect of RPSE was further investigated.

### 3.3. RPSE Reduced Pro-Inflammatory Cytokines without Hepatotoxicity in the ObD Mice

Plasma concentrations of ALT and AST were examined as indicators of liver function test (Table 3). The levels of ALT and AST in all groups were within normal ranges (<100 IU/L) [38,39] and showed no significant differences between the *db/db* and RPSE-treated group. In a previous study, the capsicoside G-rich fraction from pepper did not adversely affect hepatotoxicity in diet-induced obese mice [35]. As such, the concentration of RPSE, applied in the current study, does not either improve or damage liver function in ObD mice.

To assess the effects of RPSE on inflammatory responses, we further examined the plasma levels of pro-inflammatory cytokines such as TNF-α and IL-6 (Table 3). The *db/db* control remarkably increased the levels of TNF-α and IL-6 compared to the *db*/+, and these unfavorable phenotypes were rescued in metformin-treated mice (*p* < 0.001). The supplementation of RPSE notably decreased the level of TNF-α by 22% compared to that in the *db/db* control (*p* < 0.001). The level of IL-6 in plasma was decreased by 21% by RPSE supplementation but was not significant. Similar to these findings, red pepper extract inhibited the production of nitric oxide and TNF-α induced by lipopolysaccharide/interferon-γ (IFN-γ)-stimulated macrophages in vitro and also reduced inflammation in mice [40]. In addition, red pepper improved antigen-induced inflammation in rats, followed by the inhibition of pro-inflammatory cytokine production at the inflammation site. Capsaicin was presented as a bioactive compound in this study [41]. Taken together, these findings indicate that RPSE does not have a toxic effect that exacerbates liver damage; rather, RPSE has anti-inflammatory potential in ObD mice.

### 3.4. RPSE Improved Glucose Uptake by Amending Insulin Sensitivity in ObD Mice

**Figure 1:**
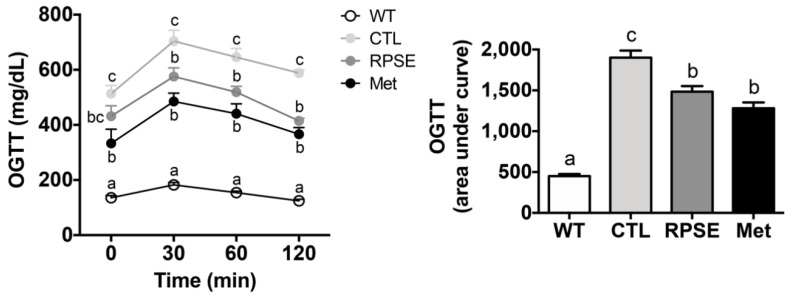
Red pepper seed extract (RPSE) decreased the area under the curve during oral glucose tolerance test in *db/db* mice. *db*/+, heterozygote mice; CTL, *db/db* mice treated with vehicle; RPSE, *db/db* mice treated with RPSE 200 mg/kg bw; Met, *db/db* mice treated with metformin 150 mg/kg bw. Values are expressed as mean ± SEM (*n* = 8). ^a–c^ Means in the row indicated by different alphabets are significantly different between groups (*p* < 0.05).

Given that hyperglycemia was rescued by RPSE supplementation, we assessed whether this benefit of RPSE is dependent on gaining insulin sensitivity (Figure 1). For OGTT, glucose bolus (2 g/kg bw) was administered by oral gavage to mice after 4 h of fasting, and blood glucose was measured at 30, 60, and 120 min post-administration. In the *db*/+ mice, the concentration of blood glucose reached a peak at 30 min post-administration and returned to a normal level within 2 h (*p* < 0.001). This result indicated that *db*/+ mice sustained sufficient glucose homeostasis. In contrast, the *db/db* control showed the highest glucose levels throughout the measuring points, which meant a successful induction of T2DM insulin resistance. The comparative drug metformin recovered insulin sensitivity i.e., postprandial glucose levels were remarkably decreased throughout measuring points, and the AUC was significantly decreased by 33% compared to the control (*p* < 0.001).

Strikingly, RPSE-treated mice showed a dramatic 30% decrease in postprandial blood glucose levels (at 120 min) compared to those in the *db/db* control (*p* < 0.001). The diminished AUC further proved that RPSE supplementation enhanced insulin sensitivity, and the degree of reduction was even similar to metformin (*p* < 0.001). As such, improved glucose uptake by RPSE supplementation in the ObD model was an innovative discovery. We previously demonstrated that 200 µg/mL of RPSE activated the translocation of GLUT4 to the plasma membrane followed by increasing glucose uptake in the C2C12 murine myotubes [19]. Similarly, there was a previous report that red pepper powder extracted using 70% ethanol improved insulin sensitivity and glycogen storage in liver and muscle tissues of ovariectomized rats fed a HFD [42]. Our findings suggested that RPSE has the ability to improve glucose uptake and insulin sensitivity in ObD mice.

### 3.5. RPSE Downregulated Hepatic Gluconeogenic Genes and Proteins via Phosphorylating FOXO1 and AMPK

**Figure 2:**
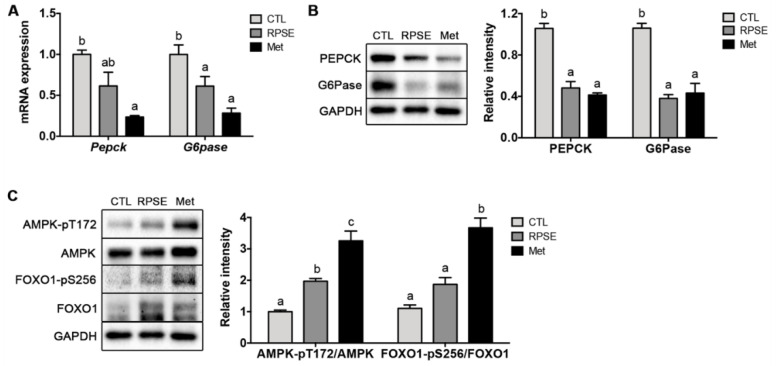
Red pepper seed extract (RPSE) improved the gene and protein expression related to hepatic gluconeogenesis in *db/db* mice. (**A**) mRNA expression of *Pepck* and *G6Pase*. (**B**) representative immunoblotting of PEPCK and G6Pase. (**C**) Representative immunoblotting of AMPK and FOXO1. The relative intensities by densitometry are shown. Expression levels were normalized to GAPDH. CTL, *db/db* mice treated with vehicle; RPSE, *db/db* mice treated with RPSE 200 mg/kg bw; Met, *db/db* mice treated with metformin 150 mg/kg bw. Values are expressed as mean ± SEM (*n* = 4). ^a–c^ Means in the row indicated by different alphabets are significantly different between groups (*p* < 0.05). AMPK, AMP-activated protein kinase; FOXO1, forkhead box protein O1; PEPCK, phosphoenolpyruvate carboxykinase; G6Pase, glucose 6-phosphatase; GAPDH, glyceraldehyde 3-phosphate dehydrogenase.

We further investigated the feature of transcriptional regulation by RPSE. First, the mRNA expression of *G6pase* and *Pepck*, the key genes encoding the rate-limiting enzymes for gluconeogenesis, was examined in RPSE-treated ObD mice (Figure 2A). Notably, the mRNA expression of *G6pase* was remarkably suppressed by 39% in RPSE-treated mice compared to the control (*p* < 0.001). The mRNA expression of *Pepck* was 38% less than that of controls in the RPSE-treated mice; however, statistical significance was not verified. The protein levels PEPCK and G6Pase encoded by the preceding genes were evaluated (Figure 2B). The protein levels were eliminated by RPSE supplementation; PEPCK and G6Pase were reduced by 43% (*p* < 0.001) and 78% (*p* < 0.01), respectively, in ObD mice. Our previous in vitro study also demonstrated that RPSE reduced gluconeogenesis by inhibiting PEPCK and G6Pase proteins in AML12 murine hepatocytes [19]. These novel findings proved that RPSE supplementation transcriptionally downregulated the expression of gluconeogenic genes and proteins, including PEPCK and GP6Pase, in the liver of ObD mice.

To identify the signal transduction pathways of gluconeogenic genes and proteins, we explored the features of FOXO1 and AMPK (Figure 2C). FOXO1 is a highly conserved forkhead family transcription factor and is involved in PEPCK and G6Pase expression. FOXO is strongly regulated by insulin through the phosphoinositide-3 kinase (PI3K)/AKT signaling pathway [43]. In the sufficient nutrients state, insulin activates AKT phosphorylation at the threonine site, followed by phosphorylating FOXO at serine and threonine sites. Such phosphorylation of FOXO1 leads to its nuclear exclusion and cytosolic retention, followed by the inhibition of FOXO1-dependent transcription of PEPCK and G6Pase [44]. Limited reports suggest that RPSE modifies the phosphorylation or translocation of FOXO1. Only our prior study has proven that RPSE activates AKT/FOXO1 to suppress gluconeogenesis in AML12 hepatocytes. Interestingly, in the present study, the phosphorylation of FOXO1 at serine 256, mediated by AKT, was increased by 180% in the RPSE-supplemented group compared to the *db/db* control. These findings suggest that the increase in phosphorylation of FOXO1 by RPSE is the dominant mechanism that explains the improved fasting glucose levels and insulin sensitivity in ObD mice.

In addition to FOXO1 phosphorylation, the impact of RPSE on AMPK, another major regulator of gluconeogenesis, was investigated. In the fasting state, AMPK phosphorylates and excludes class IIa histone deacetylases (HDAC) that interfere with the translocation of FOXO into the nucleus, resulting in the inhibition of gluconeogenesis [45]. AMPK also suppresses gluconeogenesis by inhibiting transcription factors such as hepatic nucleotide factor 4 (HNF4) and CRB-regulated transcription activator 2 (CRTC2), which promote PEPCK and G6Pase [46,47]. In the present study, RPSE-treated mice showed a 196% increase of phosphorylation AMPK at threonine 172, which accounts for AMPK activation. Previous studies reported that capsaicin from red pepper activated AMPK through the calcium/calmodulin-dependent protein kinase kinase β (CaMKKβ) [48,49] in HepG2 hepatocytes. Another study reported that ethanol extract of red pepper showed anti-diabetic activity via AMPK and PPAR-γ activation in C2C12 myotubes [50]. These prior studies certainly differ from our study with respect to the extraction solvent and biologically active compounds. Therefore, our study proved that the supplementation of RPSE stimulated AMPK activation in ObD mice. Taken together, RPSE enhanced the phosphorylation of FOXO1 and AMPK, which underlies RPSE-induced inhibition of hepatic gluconeogenesis.

## 4. Conclusions

RPSE supplementation did not alter body weight, fat accumulation, or liver damage in ObD mice. Interestingly, RPSE supplementation ameliorated the biomarkers of glycemic control, including fasting glucose, HbA1c, and insulin. RPSE supplementation also reduced pro-inflammatory cytokines and triglyceride in the blood. In addition, RPSE improved glucose uptake and insulin sensitivity according to the OGTT. RPSE supplementation transcriptionally downregulated the expression of gluconeogenic genes and proteins, including PEPCK and GP6Pase in the liver. An increased phosphorylation of FOXO1 and AMPK was identified as the underlying mechanism of the RPSE-induced anti-diabetic effect. In conclusion, these findings suggest that RPSE enhanced glycemic control by inhibiting hepatic gluconeogenesis via the phosphorylation of FOXO1 and AMPK in ObD mice.

## Figures and Tables

**Table 1:** Growth performance and body composition of *db/db* mice supplemented with red pepper seed extract (RPSE).

	*db*/+ ^1^	Control	RPSE	Metformin
**Growth performance**				
Initial body weight (g)	21.19 ± 0.32 ^a^	29.20 ± 0.23 ^b^	28.82 ± 0.27 ^b^	31.58 ± 2.72 ^b^
Final body weight (g)	24.74 ± 0.66 ^a^	40.85 ± 0.48 ^c^	41.30 ± 1.18 ^c^	33.95 ± 2.87 ^b^
Weight gain (g/week)	3.54 ± 0.54 ^a^	11.65 ± 0.48 ^b^	12.48 ± 1.30 ^b^	2.36 ± 5.51 ^a^
Total energy intake (kcal)	504.50 ± 2.52 ^a^	991.50 ± 2.11 ^b^	1040.00 ± 53.80 ^b^	890.70 ± 9.92 ^b^
**Body composition**				
Fat in tissue (%)	20.77 ± 1.17 ^a^	44.28 ± 0.86 ^b^	45.29 ± 1.74 ^b^	40.38 ± 6.89 ^b^
Lean mass (%)	77.26 ± 1.12 ^a^	67.09 ± 0.82 ^b^	64.89 ± 1.58 ^b^	66.00 ± 0.95 ^b^
Bone mineral density (mg/cm^2^)	86.85 ± 0.57	82.02 ± 1.76	89.04 ± 3.16	84.87 ± 7.76

^1^*db*/+, heterozygote mice; Control, *db/db* mice treated with vehicle; RPSE, *db/db* mice treated with RPSE 200 mg/kg bw; Metformin, *db/db* mice treated with metformin 150 mg/kg bw. Values are expressed as the mean ± SEM (*n* = 8). ^a–c^ Means in the row not sharing a common letter are significantly different between groups (*p* < 0.05).

**Table 2:** Type 2 diabetes mellitus biomarkers in the plasma of *db/db* mice supplemented with red pepper seed extract (RPSE).

	*db*/+ ^1^	Control	RPSE	Metformin
Fasting blood glucose (mg/dL)	139.80 ± 6.74 ^a^	544.20 ± 20.88 ^d^	429.40 ± 22.85 ^c^	296.30 ± 46.23 ^b^
HbA1c ^2^ (%)	3.86 ± 0.30 ^a^	8.58 ± 0.41 ^c^	6.72 ± 0.45 ^b^	4.33 ± 0.67 ^a^
Insulin (ng/mL)	1.09 ± 0.04 ^a^	2.10 ± 0.08 ^c^	1.76 ± 0.08 ^b^	1.49 ± 0.09 ^b^
Triglyceride (mg/dL)	72.02 ± 4.08 ^a^	135.40 ± 9.94 ^c^	103.90 ± 3.01 ^b^	48.32 ± 6.81 ^a^
Non-esterified fatty acid (mEq/L)	0.54 ± 0.07 ^a^	1.21 ± 0.04 ^b^	0.99 ± 0.06 ^b^	0.56 ± 0.07 ^a^

^1^*db*/+, heterozygote mice; Control, *db/db* mice treated with vehicle; RPSE, *db/db* mice treated with RPSE 200 mg/kg bw; Metformin, *db/db* mice treated with metformin 150 mg/kg bw. ^2^ HbA1c, glycated hemoglobin. Values are expressed as the mean ± SEM (*n* = 8 per group).^a–d^ Means in the row not sharing a common letter are significantly different between groups (*p* < 0.05).

**Table 3:** Hepatic function and the pro-inflammatory cytokines in *db/db* mice.

	*db*/+ ^1^	Control	RPSE	Metformin
ALT ^2^ (IU/L)	37.35 ± 6.14	45.40 ± 5.00	40.81 ± 2.69	46.31 ± 2.64
AST (IU/L)	33.17 ± 3.20 ^a^	46.29 ± 4.80 ^ab^	49.45 ± 5.39 ^b^	38.47 ± 3.41 ^ab^
TNF-α (pg/mL)	94.39 ± 8.13 ^a^	188.20 ± 11.79 ^c^	146.70 ± 7.44 ^b^	119.60 ± 11.52 ^ab^
IL-6 (pg/mL)	62.33 ± 4.52 ^a^	164.10 ± 15.24 ^b^	129.10 ± 11.28 ^b^	73.84 ± 9.21 ^a^

^1^*db*/+, heterozygote mice; Control, *db/db* mice treated with vehicle; RPSE, *db/db* mice treated with red pepper seed extract 200 mg/kg bw; Metformin, *db/db* mice treated with metformin 150 mg/kg bw. ^2^ ALT, alanine aminotransferase; AST, aspartate transaminase; TNF-α, tumor necrosis factor-alpha; IL-6, interleukin 6. Values are expressed as the mean ± SEM (*n* = 8). ^a–c^ Means in the row not sharing a common letter are significantly different between groups (*p* < 0.05).
